# Body Image Disturbances and Weight Bias After Obesity Surgery: Semantic and Visual Evaluation in a Controlled Study, Findings from the BodyTalk Project

**DOI:** 10.1007/s11695-020-05166-z

**Published:** 2021-01-06

**Authors:** Paolo Meneguzzo, Simone Claire Behrens, Angela Favaro, Elena Tenconi, Vincenzo Vindigni, Martin Teufel, Eva-Maria Skoda, Marion Lindner, M. Alejandra Quiros-Ramirez, Betty Mohler, Michael Black, Stephan Zipfel, Katrin E. Giel, Chiara Pavan

**Affiliations:** 1 Department of Neuroscience, University of Padova, via Giustiniani 2, 35128 Padova, Italy; 2 Department of Psychosomatic Medicine and Psychotherapy, Medical University Hospital Tübingen, Tübingen, Germany; 3 Max Planck Institute for Intelligent Systems, Tübingen, Germany; 4 Padova Neuroscience Center, University of Padova, Padova, Italy; 5 Clinic for Psychosomatic Medicine and Psychotherapy, University of Duisburg-Essen, LVR University-Hospital Essen, Essen, Germany; 6 Psychology Department, University of Konstanz, Konstanz, Germany; 7 Department of Medicine, University of Padova, Padova, Italy

**Keywords:** Body image, Obesity, Obesity surgery, Weight bias, Body weight dissatisfaction

## Abstract

**Purpose:**

Body image has a significant impact on the outcome of obesity surgery. This study aims to perform a semantic evaluation of body shapes in obesity surgery patients and a group of controls.

**Materials and Methods:**

Thirty-four obesity surgery (OS) subjects, stable after weight loss (average 48.03 ± 18.60 kg), and 35 overweight/obese controls (MC), were enrolled in this study. Body dissatisfaction, self-esteem, and body perception were evaluated with self-reported tests, and semantic evaluation of body shapes was performed with three specific tasks constructed with realistic human body stimuli.

**Results:**

The OS showed a more positive body image compared to HC (*p* < 0.001), higher levels of depression (*p* < 0.019), and lower self-esteem (*p* < 0.000). OS patients and HC showed no difference in weight bias, but OS used a higher BMI than HC in the visualization of positive adjectives (*p* = 0.011). Both groups showed a mental underestimation of their body shapes.

**Conclusion:**

OS patients are more psychologically burdened and have more difficulties in judging their bodies than overweight/obese peers. Their mental body representations seem not to be linked to their own BMI. Our findings provide helpful insight for the design of specific interventions in body image in obese and overweight people, as well as in OS.

## Background

A significant proportion of patients with obesity surgery (20–30%) experience an unfavorable outcome within the first postoperative years [1], and successful weight loss is not necessarily a predictor of improvement of the psychosocial outcome [2]. A potential mechanism could be body image, which not only drives approximately 30% of individuals’ choices about food consumption [2–4] but also includes cognitions and attitudes about the self and how it relates to social standards. In this point of view, patients seeking contouring surgery represent an interesting population due to their exposure to body changes and the needing of specific interventions on body image [5, 6].

Body image has been defined as the multidimensional construct derived from the perceptions, thoughts, and feelings associated with the body and bodily experiences [7, 8]. Body image is not only the satisfaction/dissatisfaction with one’s body; it also has a cognitive component that reflects the relationship between the evaluation of body shapes and the positive/negative valence attributed to them [9]. Body judgments are characterized by increasing rates of prejudice and discrimination toward overweight people, even if there is an increasing rate of overweight in the population [10]. Mental health is compromised by negative body image aspects, with an increase of psychopathological symptomatology, from depression to eating dysfunctional behaviors [11, 12].

Obese and overweight people are more exposed to negative evaluations of their body and their selves, with dramatic impacts on their everyday lives, especially in their perceptions of sizes, spaces, and attitudes [13–15]. Moreover, even though obese patients often experience an improvement of their body images after obesity surgery [16], patients could suffer the consequences of the internalization of weight stigmatization with weight gain or treatment drop out [17–20]. Indeed, obese and overweight patients exhibit specific dysfunctional cognitive body schemata that are not easily detachable from body image construct, and that are different from the normal weight population ones [21]. An implicit or explicit weight stigmatization could bring people to feel more “disgust,” “blame,” or “contempt” for overweight bodies—both their own bodies and others—with an influence on behaviors and mental health [22–25]. Such effects could interfere with improving body image after obesity surgery, and, in a second step, reduce mental health and thereby compromise the weight loss [19].

Excess skin cannot be reduced with physical activities [26], and on top of the nursing challenges, it constantly reminds a patient to the fact that he or she has been extremely obese. Literature has shown that even after contouring surgery, patients showed a low satisfaction with their own body, with a significant role of expectation on the judgment of the results [27, 28]. However, it so far remains open how these patients actually perceive their body as compared to other bodies, and how their body image relates to the body image of people who lack experience of obesity surgery.

To meet this question, this study takes a comprehensive approach to investigate body image of obesity surgery patients looking for a contouring surgery. Using established psychometric instruments as well as computerized experimental approaches, different facets of body image construct have been evaluated, comparing overweight-obese people with and without a history of obesity surgery. Our hypothesis is that OS patients could show an impaired body image evaluation and cognitive representation, due to their history of significant weight fluctuations. Moreover, different semantic evaluation of body shapes could be found between included samples, which could be a possible target of intervention.

## Methods

### Participants

Thirty-four people (2 male, 32 female) with previous obesity surgery interventions for severe obesity seeking a contouring surgery were recruited in the outpatient service of the Plastic Surgery Unit of the University of Padova. Obesity surgery patients were recruited between 2 and 10 years after their laparoscopic sleeve gastrectomy; they all had stable weights for, at least, 6 months. A group of 35 BMI-matched controls (MC) of matched age and BMI were selected from a sample of international subjects who participated in a research project about body image evaluation (3 male, 32 female), called BodyTalk project [29]. The inclusion criteria for both groups were an age between 18 and 65 years and no severe mental and medical comorbidity (e.g., no eating disorders), no neurological trauma and disorders, nor drug addictions. The exclusion criteria for the MC were that they could not have a history of obesity surgery interventions or desire to have any. Informed consent was collected from each participant. The study was approved by the local ethic committee as part of a larger study on the cognitive evaluation of obesity surgery patients, and it complies with the provisions of the Declaration of Helsinki.

### Assessment Instruments

OS participants were evaluated by a trained researcher with clinical interviews, assessing weight history, behaviors and previous psychological and surgery interventions, and exclusion and inclusion criteria were applied. After providing informed consent, included participants were administered specific questionnaires about eating and weight concerns, depression, self-esteem and body evaluation, and afterwards completed three computerized tasks. The MC were tested with the same tasks using the same methodology across the different centers. The following instruments were used.

The Rosenberg self-esteem scale (RSES) [30] is a well-established self-reported 10-item test about self-esteem. For each item, there is a Likert scale response from “strongly agree” to “strongly disagree.” Results higher than 15 may indicate seriously low self-esteem.

The physical appearance comparison scale (PACS) [31] is a 5-item self-reported test used to assess the degree of physical comparison with others in various social situations. Responses are collected on a Likert scale, ranging from “never” to “always.” Higher scores indicate higher social comparison tendencies.

The body dissatisfaction subscale and the drive to thinness subscale of the eating disorder inventory (EDI) [32] are both well-established measures of specific psychopathology constructs about body shapes. Each item is on a 6-point scale, ranging from “always” to “never” and rated 0–3, and higher scores indicate higher body dissatisfaction.

The patient health questionnaire (PHQ-9) [33] is a 9-item self-reported test, and it is considered a good measure of depression. There are four answer categories from “not at all” to “almost every day”, and higher total scores indicate higher depression symptomatology.

The body image questionnaire (BIQ-20) [34] is a measure of the dynamism of one’s body (e.g., “I feel very fit”—perception of body dynamics (PBD) subscale), as well as of its rejection (e.g., “My body often annoys me”—negative evaluation of the body (NEB) subscale). There are five answer categories, ranging from “Not true” to “Absolutely true,” and higher scores indicate a more negative body image. The translation from the original Germany version to the Italian version was performed independently by an author and a professional translator, and the two versions were reviewed by two different German-speaking authors. A backward translation was performed in order to evaluate the semantic value of the questionnaire. Good reliability was found: PBD Cronbach’s α = 0.81 and NEB Cronbach’s α = 0.85.

### Study Design

**Fig. 1:**
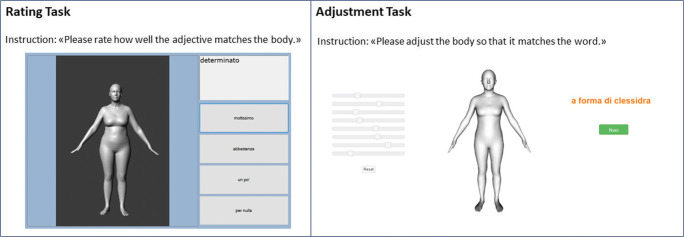
Illustration of the two computerized tasks that were used for the assessment. The rating test consisted of 12 different bodies and 16 adjectives. The adjustment task was performed for the 16 adjectives and then for the visual representation of the patient’s or control’s own body. The order of the bodies/adjectives was randomized

Semantic evaluation of body shapes and weight bias were assessed using a computerized evaluation of body shapes, as already applied to other clinical population [29]. The body shape evaluation was composed of three different tasks performed by participants and recorded using a 17″ laptop in the morning. See Fig. 1 for a visual representation.

1. In the first task, the *rating task*, 12 body images, and 16 adjectives were presented to participants and asked to evaluate how the match was adequate. The image set was composed of a realistic human body stimulus of different weights: underweight, normal weight, overweight, and obese [35]. We selected the bodies to cover a range in BMI from 15.5 to 36.5 kg/m^2^. The adjective set was composed of 16 adjectives describing both physical and behavioral factors selected from literature about weight bias and already used in other studies: active, apple-shaped, attractive, clumsy, determined, feminine, heavy-set, hourglass-shaped, impulsive, insecure, lazy, open-minded, pear-shaped, smart, thin, and unfriendly [36]. The adjectives were translated into Italian by a professional translator and an author independently and then checked by another author to evaluate the differences. A third author then translated back the adjectives in order to evaluate semantic correspondence. All possible combinations were presented randomly to the participants, and the evaluation was performed with a 4-point Likert scale from “very much” to “not at all.”
2. In the second task, the *adjustment task*, an adapted version of the body shape visualization tool, was used [36]. The participants were asked to modify a digital, realistic neutral human presented on the computer screen using eight scrollbars, each representing a principal component of the body. The goal of this task was to generate a biometrically plausible 3D body model for each specific adjective from the adjective set. The first trial was carried out without providing any adjectives, and it was used to familiarize the participants with the scrollbars. Subsequently, each adjective from the first task set was presented randomly, and participants were asked to generate a prototypic body matching the adjective. At the end of the task, participants were asked to reproduce their own body.
3. In the third task, the *valence assessment task*, people were asked to evaluate all the presented adjective with a 5-point Likert scale from “clearly negative” to “clearly positive,” as a measurement of adjectives valence. Adjectives were presented to all groups in a randomized order.

### Statistical Analysis

The analysis was conducted with IBM SPSS Statistics 25.0 (SPSS, Chicago, IL, USA). A normal distribution test was performed for all variables. The demographic variables and questionnaire scores were tested using independent t-tests. To analyze semantic evaluation of body shape, we estimated the BMI of the generated bodies and compared them between the groups.

In the adjustment task, the height, in meters, of the human model was calculated subtracting its lowest point from its highest point. The volume in cubic meters of the models was calculated as described by Zhang and Chen [37], and, dividing its volume by the average human body density (1010 kg/m^3^) [38] was obtained the weight of the models and was calculated the BMI.

In the valence assessment, no differences between subgroups emerged in the analysis of the assigned adjective valence (results from the valence assessment task). Adjectives were then grouped by the valences, from negative to positive according to participants’ ratings, and the average BMIs were evaluated in order to look at the mental representation of body shapes.

To evaluate weight bias, data from the rating task were aggregated with data from the valence assessment task. Thus, we obtained the valence values for body weight categories (i.e., underweight, normal weight, overweight, and obese) and we were able to split them into positive/negative adjectives. The normally distributed data was analyzed using *t* tests. Pearson’s correlation analysis was used to evaluate how the BMI of rated body shapes was associated with valence of the adjective. Comparisons between correlations were performed using the Fisher’s r to z transformation [39].

Body size estimation accuracy was assessed using the body perception index. It was calculated as the percentage of the ratio between the estimated BMI through the visualization tool and the actual BMI, in order to compare body perception [40]. Linear regression analysis were performed between actual BMI and own represented BMI, with the goal to evaluate if the accuracy could be explained by the participants’ BMI.

Several regression analyses were also performed between the human model’s BMI and psychological scores with stepwise approaches. The alpha was set at 0.05 for all the analyses. The effect sizes were calculated with Cohen’s delta.

## Results

A summary of the demographic characteristics and questionnaire scores of each group can be found in Table 1. Levels of self-esteem, body dissatisfaction, drive for thinness, depression, and body perception were found to be significantly different in the two investigated groups. The OS patients and MC had similar BMIs, but OS had a significantly higher lifetime maximum weight than MC (*p* < 0.001). The OS patients reported a less negative body dissatisfaction than MC with EDI subscales (*p* = 0.001), but no difference was recorded about negative body evaluation using the BIQ-20 scale between the two groups, even if OS patients perceived their body as less dynamic (*p* = 0.002). Moreover, OS patients showed lower self-esteem (*p* < 0.001) and more depressive symptoms (*p* = 0.019) than control peers.

**Table 1:** Demographic characteristics of the included samples

	OS (SD) *n* = 34	MC (SD) *N* = 35	*t*	*p*	*d*
Age, years	48.48 (12.60)	42.43 (14.50)	1.833	0.071	0.445
Women, %	96.88	94.44			
BMI, kg/m^2^	29.33 (5.02)	32.83 (13.52)	− 1.396	0.160	0.343
BMI max lifetime, kg/m^2^	46.43 (8.81)	34.56 (14.09)	− *4*.*212*	< *0*.*001*	1.010
BMI min after puberty, kg/m^2^	24.62 (5.65)	22.24 (4.24)	− 1.979	0.052	0.476
RSES	17.21 (2.06)	21.26 (4.54)	− *4*.*780*	< *0*.*001*	1.148
PACS	13.73 (4.66)	14.66 (3.15)	− 0.969	0.336	0.234
EDI-2 Drive for thinness	7.56 (5.36)	14.32 (12.70)	− *2*.*896*	*0*.*006*	0.693
EDI-2 Body dissatisfaction	10.27 (6.56)	22.71 (18.83)	− *3*.*678*	*0*.*001*	0.882
PHQ9	7.06 (4.13)	5.06 (2.61)	*2*.*405*	*0*.*019*	0.579
BIQ-PBD	30.52 (4.93)	35.04 (6.48)	− *3*.*229*	*0*.*002*	0.785
BIQ-NEB	29.70 (3.90)	27.89 (10.46)	0.935	0.353	0.229

*SD* standard deviation, *OS* obesity surgery subjects, *MC* BMI-matched control, *BMI* body mass index, *RSES* Rosenberg Self-Esteem Scale, *PACS* Physical Appearance Comparison Scale, *EDE*-*Q* Eating Disorder Examination Questionnaire, *PHQ* Patient Health Questionnaire, *BIQ* body image questionnaire, *PBD* perception of body dynamics subscale, *NEB* negative evaluation of the body subscale

**Fig. 2:**
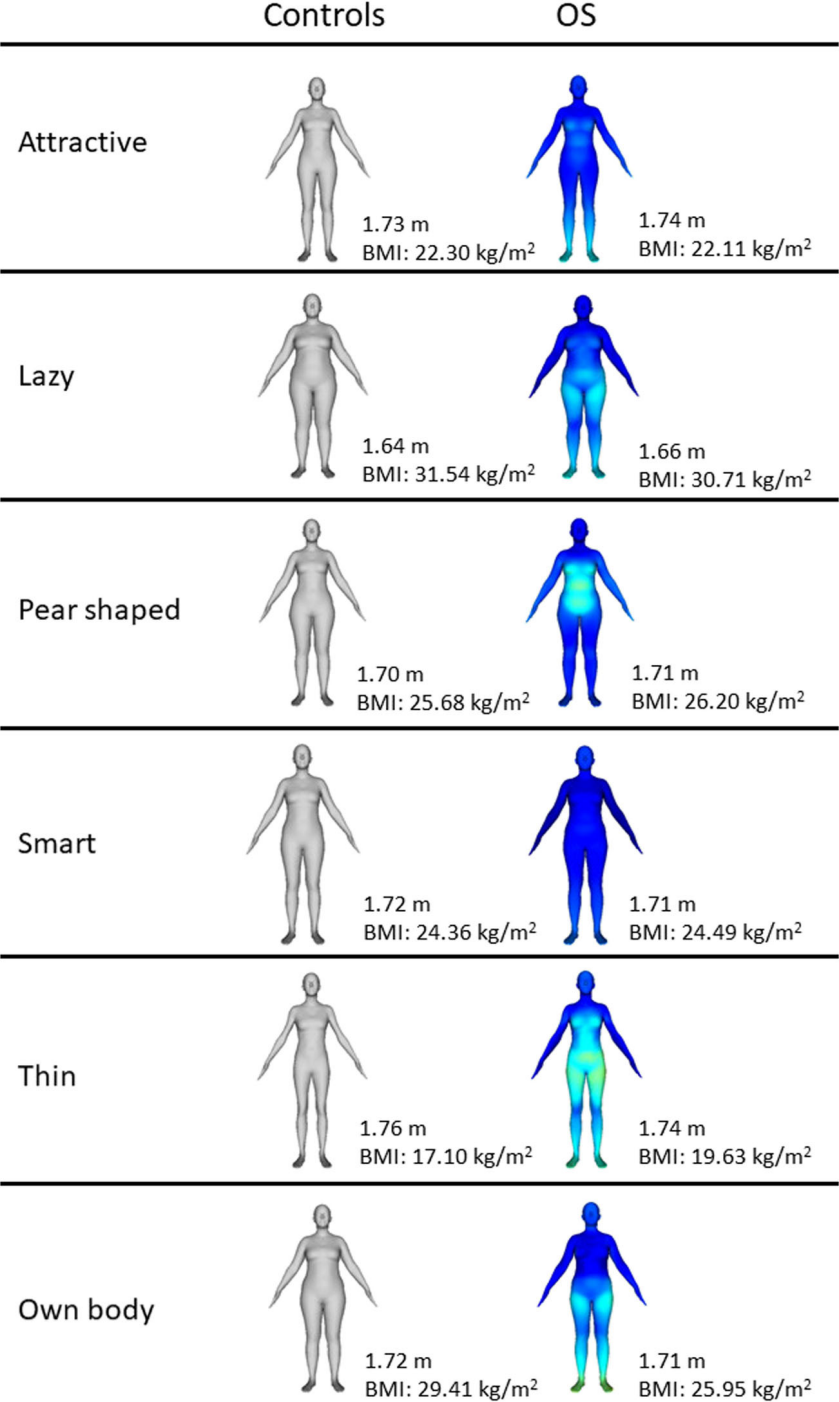
Visual representation of the adjustment task. The height and the BMI are reported for each figure. Colors indicate the difference between control’s body model and OS’ one: dark blue represents no differences, lighter colors showed more difference

The analysis of the semantic evaluation of body shape (adjustment task) suggested the presence of very similar specific body shape images linked to specific adjectives in both two groups. The 3D body shape models’ BMI are reported in Table 2. Only for specific adjectives (i.e., heavy-set and thin), the analysis have reported different BMI between OS patients and MC. Looking at specific adjectives, the analysis of the modification of the body components showed that between OS and controls different body parts are differently adjusted, even if the final BMIs are the same, see Fig. 2 for a visual representation of adjectives. For example, it is possible to evaluate that comparing OS and controls, the body model for “thin” was quite globally modified differently by OS that adjusted a fuller body, and, instead, for “lazy” the OS patients create a body model with a similar but with different hips and abdomen. Moreover, looking at the “own body” models, the OS patients showed a greater focused about their waists than the controls.

**Table 2:** BMI of realistic modified human bodies in the adjustment task

	OS mean (SD)	MC mean (SD)	*t*	*p*	*d*
Active	22.39 (3.10)	21.28 (2.77)	1.478	0.144	0.378
Apple shaped	32.07 (5.27)	33.28 (5.99)	− 0.851	0.398	0.214
Attractive	22.11 (2.88)	22.30 (2.83)	− 0.260	0.796	0.067
Clumsy	30.44 (5.18)	29.11 (6.04)	0.940	0.351	0.281
Determined	23.86 (3.30)	22.18 (2.73)	*2*.*168*	*0*.*034*	0.555
Feminine	21.81 (3.35)	22.20 (2.94)	− 0.476	0.636	0.124
Heavy set	30.25 (4.10)	34.89 (5.90)	− *3*.*678*	*0*.*000*	0.913
Hourglass shaped	24.50 (4.32)	23.62 (5.21)	0.733	0.466	0.184
Impulsive	24.49 (3.14)	26.11 (4.63)	− 1.589	0.119	0.410
Insecure	29.75 (5.09)	29.14 (5.76)	0.446	0.657	0.112
Lazy	30.71 (5.03)	31.54 (6.66)	− 0.550	0.585	0.141
Open minded	25.88 (3.22)	24.18 (2.21)	*2*.*371*	*0*.*021*	0.616
Pear shaped	26.20 (3.49)	25.68 (3.91)	0.555	0.581	0.140
Smart	24.49 (2.00)	24.36 (2.00)	1.207	0.233	0.065
Thin	19.63 (3.24)	17.10 (1.92)	*3*.*852*	*0*.*000*	0.950
Unfriendly	24.01 (4.15)	22.93 (6.48)	0.769	0.446	0.198
Own body	25.95 (4.37)	29.41 (6.98)	− *2*.*294*	*0*.*027*	0.594

*OS* obesity surgery subject, *MC* BMI-matched control, *BMI* body mass index, *SD* standard deviation

The assessment of weight bias through a correlation analysis between the BMI of the generated body shapes and adjective rating are reported in Table 3. This analysis suggests that participants were more willing to accept positive adjectives as a match when the BMI was low, and assigned negative adjectives as a match when the BMI was high. Both groups showed the same trends for a weight bias, even though the correlation factors exhibited different associations between BMI and valences. Comparisons between correlation coefficients (Fisher’s r to z transformation) showed no significant differences (negative adjectives: *Z* = 0.244, *p* = 0.807; neutral: *Z* = − 1.068, *p* = 0.286; positive: *Z* = − 0.262, *p* = 0.793).

**Table 3:** Correlations analysis between valences and BMI of the rating task

	Adjective rating	Avatar BMI
OS	Negative	.337**
	Neutral	.087**
	Positive	− .295**
MC	Negative	.281**
	Neutral	.344**
	Positive	− .233**

*OS* obesity surgery subject, *MC* BMI-matched control, *BMI* body mass index; **p < .001

The results from the modified own body shape were grouped according to the adjective valences (see Table 4). This grouping revealed how the represented BMIs exhibited differences between the OS and MC participants only with positive adjectives. Moreover, the OS participants modified the human body model building higher BMI.

**Table 4:** Realistic human BMI and attributed valences, an integration of the adjustment, and valence tasks results

	Valence (SD)		OS (SD)	MC (SD)	*t*	*p*	*d*
Unfriendly	− 1.56 (0.60)	Clearly negative	24.01 (4.15)	22.93 (6.48)	0.769	0.446	0.198
Insecure	− 1.07 (0.61)	Rather negative	30.74 (5.16)	30.77 (6.29)	− 0.035	0.972	0.006
Lazy	− 1.02 (0.66)						
Clumsy	− 0.85 (0.66)						
Apple shaped	− 0.63 (0.76)						
Heavy set	− 0.33 (0.89)	Neutral	26.36 (4.32)	27.58 (6.55)	− 1.683	0.094	0.219
Pear shaped	− 0.31 (0.82)						
Impulsive	− 0.19 (0.80)						
Hourglass shaped	0.02 (1.02)						
Thin	0.65 (0.87)	Rather positive	21.96 (3.43)	21.01 (3.30)	*2*.*485*	*0*.*013*	0.282
Active	1.33 (0.61)						
Attractive	1.37 (0.68)						
Feminine	1.41 (0.63)						
Determined	1.43 (0.60)						
Open minded	1.52 (0.69)	Clearly positive	25.19 (2.75)	23.97 (2.46)	*2*.*591*	*0*.*011*	0.468
Smart	1.63 (0.71)						

*OS* obesity surgery subject, *MC* BMI-matched control, *SD* standard deviation

In this table, adjectives has been aggregated based on the average valences attributed and the average adjustment BMI of the virtual body model has been calculated for each subgroups

Moreover, the own body assessments of the OS patients were significantly different for the real BMI and reconstructed BMI (*t*(33) = 3.414, *p* = 0.002, *d* = 0.786), but for the MC, the difference between the assessments of the two BMI categories was only weakly significant (*t*(33) = 2.072, *p* = 0.050, *d* = 0.368). Body perception indices in OS patients results 89.62 (± 18.24), and in MC it was 86.28 (± 21.18), with a non-significant difference between them (*t*(62) = − 0.679, *p* = 0.500).

**Fig. 3:**
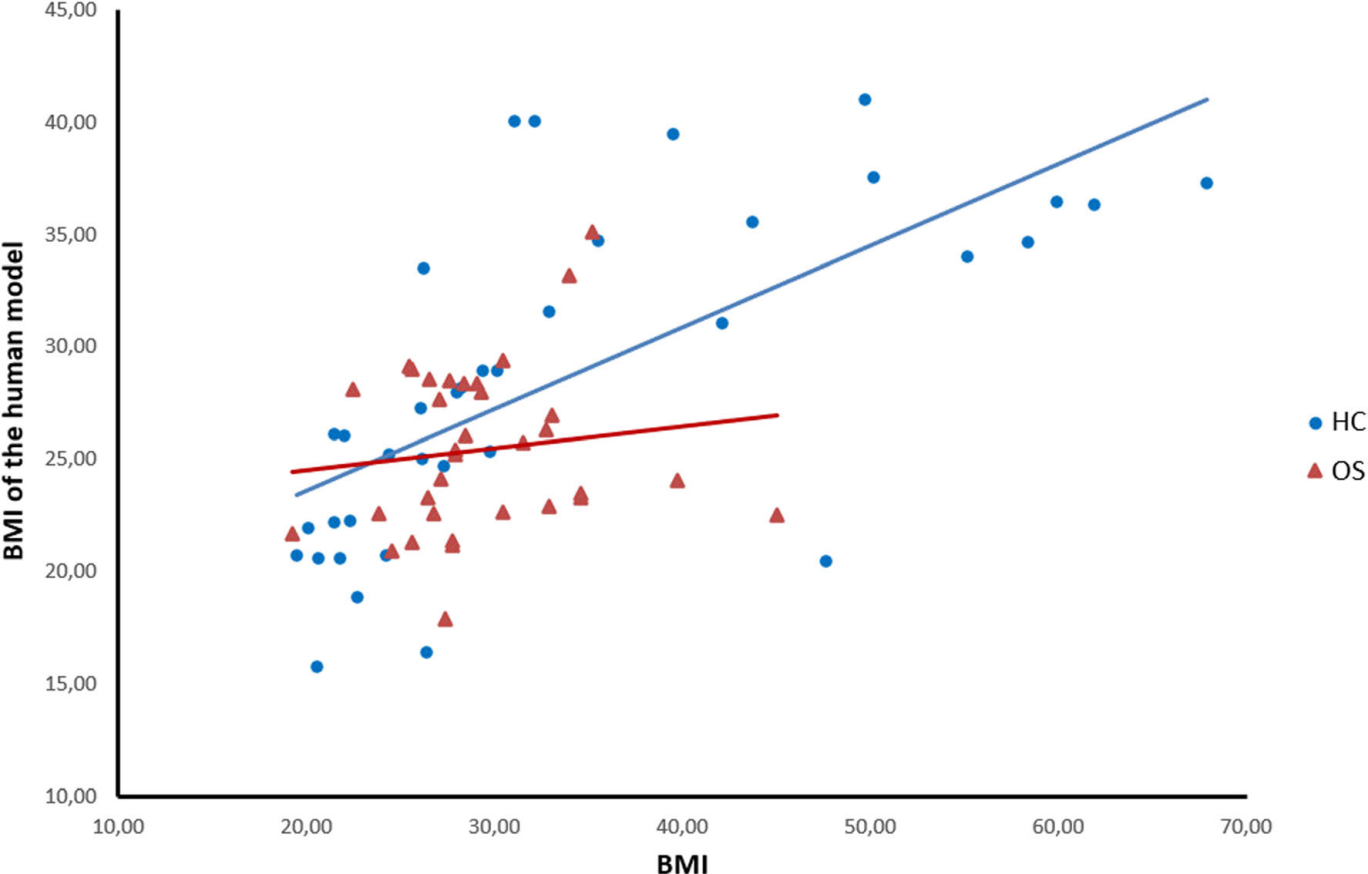
The relationship between the actual BMI and the “own body” human model BMI by the adjustment task. The graphs show that a relationship between these two variables is maintained only in MC, and this could be interpreted as an impaired ability for OS to update their own body image

Regression analysis showed that between actual BMI and own represented BMI, there is no relationship in OS patients (*F*(1,32) = 0.549, *p* = 0.464), but it is present in MC (*F*(1,33) = 26.692, *p* < 0.001, *R*^2^ = 0.447). While in OS patients, actual BMI was not associated with represented BMI, MC tended to adjust the represented own body to higher BMI the higher their actual BMI was. See Fig. 3 for a graphical representation. Looking at relationship between represented own BMI and psychological features, no significant relationship has been found neither for OS nor for MC.

## Discussion

Body image and shape evaluation is an important aspect in the psychopathology of severely obese patients and OS patients because it is strongly linked to intervention outcomes and quality of life [27, 41]. To gain more insights on this important topic, we conducted a semantic evaluation of body shapes with a group of OS patients looking for contouring surgery who were compared to a group of weight and age-paired MC. It has already been demonstrated that obesity surgery intervention has a positive effect on body image [19, 42], but less is known about how people describe and evaluate body shapes and weights. Overall, our results confirmed that OS patients have a more positive image of their own body, corroborating data from OS literature [42], but we also showed specific aspects of the relationship between mental body representation and real body sizes.

The OS patients had lower self-esteem and higher levels of depression than the MC. Both factors are not clinically relevant according to standard scoring [43, 44], but they confirmed that OS patients should be considered vulnerable to psychological distress [45]. The drive for thinness and body dissatisfaction are bodily psychopathologies, and our results are in line with data from literature: the OS participants had lower levels of bodily psychopathology and had the greatest weight loss [46]. Thus, even if the two groups have the same average BMI, losing weight could still reduce bodily focused psychopathology. However, OS still perceived lower body dynamics than their BMI peers, which is a construct that indicates how healthy, energetic, and confident a person feels about their body. This could be linked to the presence of excess skin that can modify the perception of one’s own body movements, and justify the perceived need for a contouring surgery. This is in line with previous literature data that have shown that, even after obesity surgery, body image and the perception of body dynamic still remains poor comparable with peers without obesity surgery [16]. It could be the result of the previous morbid obesity, as well as a new problem linked to the body change [47]. Indeed, our sample was selected in the waiting list for contouring intervention for surplus skin and presented a maximum BMI—and then body sizes—significantly higher than MC with a possible impairment of every day movements. This aspect should be evaluated in future studies, with longitudinal design.

The results from the body evaluation showed that OS patients and MC do not show different relationships between negative or positive judgments and the mental shape representations, even though OS patients associate positive adjectives with a higher BMI than that produced in the models by MC. Also, the body area that OS patients changed in the human models showed a qualitative difference with the controls, showing a focusing on their waists. Body valence and judgment is a neglected topic in the OS literature, but the data showed that, after obesity surgery and weight loss, patients desire more unrealistic body shapes and this was interpreted as a higher adherence to the western thin-ideal [19, 48]. The literature has already revealed that obesity surgery can positively change body image and body dissatisfaction [19, 49], our results added that these improvements could not be explained only by individual judgmental style about own body: severe obesity and the consequent obesity surgery seems to make the difference between people with the same BMI but with different weight and surgery history.

Our results are also in line with the literature that has demonstrated how overweight and obese people misperceived their weight, and that this aspect persists even after obesity surgery [48, 50, 51]. We do not find any difference in the underestimation of the own body sizes between subgroups, in accordance with the possible presence of a visual recalibration of the own weight status in overweight people, called as “the visual normalization theory” [52, 53]. However, in our sample, OS patients also showed a disruption of the relationship between the represented BMI and the actual BMI, which is preserved in MC, instead. Even with mirror evaluation, the literature has showed that ex-obese patients show a poorer estimation of their shapes compared to peers with the same BMI [54], as well as the impaired ability to notice weight gain in realistic 3D human models which represent themselves [55]. This impaired ability to update body image memory in OS patients could be explained by a sort of weight category bias, with OS patients that stopped to identify themselves as obese, a situation that they could linked to psychological burden situations, worsened by interpersonal problems, chronic stress, or anxiety [56]. This could have a significant implication for public health, because OS patients seem to be incapable of identifying weight changes after massive weight loss, as well as they seem to be into a weight category bias with a cognitive dissonance that could have a role in the weight reduction outcome, if it does not fit their goals [53].

However, the present findings should be viewed with caution. Our study is an observational one with a cross-sectional design, and thus, no inferences are possible about changes linked to the obesity surgery intervention. Results should be considered as preliminary due to the small sample included, that do not allow to perform a comparison between male/female. However, the selection of the MC paired to patients by age and weight should be considered a strength. Future research should be designed with a longitudinal approach with a larger sample of participants, covering the periods before and after obesity surgery and any counter-interventions.

In conclusion, our data suggest that body size representation and own body shape cognitive perception is complex, and maybe it is also a highly individual ability. Our data confirmed the impaired ability of obese and overweight patients to match actual body sizes with represented human body models, as well as showing a disrupted relationship between actual body and its mental representation in obesity surgery patients seeking for contouring surgery. Indeed, body image is an important factor for obesity surgery outcomes, especially in patients who are experiencing body changes and problematic excess skin. These results advocates for more studies about body image and body shape evaluation for people who undergo obesity surgery, as well as in overweight subjects seeking for weight interventions, because more data are needed.
